# Determinants, barriers, and facilitators of healthcare access for patients with hypertension in rural Ghana: applying the Andersen-Newman model of healthcare utilization

**DOI:** 10.1080/16549716.2025.2599567

**Published:** 2025-12-22

**Authors:** Solomon Nyame, Daniel Boateng, Keziah Opoku Marfo, Abdulaziz Mohammed Hussen, John Amoah, Kwame Adjei, Joyce Gyamfi, Martin Heine, Engelbert A. Nonterah, Diederick E. Grobbee, Olugbenga Ogedegbe, Kerstin Klipstein-Grobusch, Kwaku Poku Asante

**Affiliations:** a Kintampo Health Research Centre, Research and Development Division, Ghana Health Service, Ghana; b Julius Global Health, Department of Global Public Health and Bioethics, Julius Center for Health Sciences and Primary Care, University Medical Center, Utrecht University, Utrecht, The Netherlands; c Kwame Nkrumah University of Science and Technology, Kumasi, Ghana; d Department of Population Health, New York University School of Medicine, Center for Healthful Behavior Change, New York, NY, USA; e Institute of Sport and Exercise Medicine, Department of Exercise, Sport and Lifestyle Medicine, Faculty of Medicine and Health Sciences, Stellenbosch University, Cape Town, South Africa; f Navrongo Health Research Centre, Ghana Health Service, Navrongo, Ghana; g Department of Epidemiology, School of Public Health, CK Tedam University of Technology and Applied Sciences, Navrongo, Ghana; h Division of Epidemiology and Biostatistics, School of Public Health, Faculty of Health Sciences, University of the Witwatersrand, Johannesburg, South Africa

**Keywords:** Quality of Care for Chronic Conditions, Healthcare use, determinants, hypertension, mixed methods, Ghana

## Abstract

**Background:**

Hypertension is a major risk factor for cardiovascular diseasemorbidity and mortality, affecting 25% of adults in Ghana. Access to adequate care is critical for effective hypertension management.

**Objective:**

Evaluate healthcare utilisation among patients with hypertension and identify determinants.

**Methods:**

Guided by the Andersen and Newman model, we examined predisposing, enabling, and need factors affecting HCU. Data were collected from 600 patients with hypertension, 19 in-depth interviews with health workers, and six focus group discussions with patients. Logistic regression was used for quantitative analysis, while qualitative data were analyzed thematically.

**Results:**

In all, 73% of patients with hypertension used health care. Key predisposing factors included age 70+ years (adjusted odds ratio [aOR]: 1.97, 95% CI: 1.06–3.69) and being female (aOR: 2.32, 95% CI: 1.53–3.54). Enabling factors included health insurance (aOR: 4.07, 95% CI: 2.04–8.20), closer proximity to referral facilities (aOR: 2.28, 95% CI: 1.44–3.65), and care at district hospitals (aOR: 3.37, 95% CI: 1.94–6.03). Need factors were not associated with HCU. Barriers included financial difficulties, reliance on alternative medicines, poor health-seeking behavior, delays, erratic medication supplies, and health insurance limitations.

**Conclusions:**

This study finds high healthcare use (73%) among rural Ghanaian hypertension patients, mainly driven by demographic and structural factors. It highlights ongoing inequalities, especially among men. Interventions should focus on addressing gender issues, enhancing access to insurance, and strengthening district hospital services. Future research should evaluate the quality and consistency of hypertension care to improve health outcomes.

## Paper Context

- **Main findings:** Age and gender were the primary predisposing factors for high healthcare utilization. Additionally, access to valid health insurance, type of health facility, and proximity to healthcare facilities were the most influential enabling factors.
- **Added knowledge:** The Andersen and Newman behavioral model guided analysis of determinants linked to high healthcare utilization among patients diagnosed with hypertension in rural Ghana.
- **Global health impact for policy and action:** Evidence on healthcare utilization is crucial for healthcare providers and policymakers when developing interventions and programs to address hypertension in the community.

## Background

Hypertension is a leading risk factor for cardiovascular disease, contributing to 10.8 million deaths in 2019, with over two-thirds occurring in low- and middle-income countries (LMICs) [1,2]. In sub-Saharan Africa (SSA), the prevalence of hypertension has risen, with 48% (95% CI: 42–54%) in women and 34% (95% CI: 29–39%) in men in 2019 [3]. The burden of hypertension in Ghana is evident in healthcare utilization; in 2017, over half a million Ghanaians visited outpatient departments for hypertension, more than twofold increase since 2002 [4]. That same year, hypertension and related heart diseases were the leading causes of mortality in Ghana.

Access to regular primary care is essential for effective hypertension management [5]. Early identification through community-based screenings enables healthcare providers to intervene and proactively manage the condition [6]. However, equitable access to care depends on financial affordability, geographic proximity, availability of healthcare professionals, timely appointments, and culturally appropriate services [7]. In Ghana, several initiatives aim to address these barriers. The National Health Insurance Scheme (NHIS) was introduced to reduce financial barriers by covering healthcare costs for hypertension management [8]. In 2022, the Community Health Planning and Services (CHPS) program expanded access to care at the community level by deploying Community Health Officers (CHOs) to provide services in homes or CHPS compounds and refer individuals with hypertension for further care [9,10].

**Figure 1.**
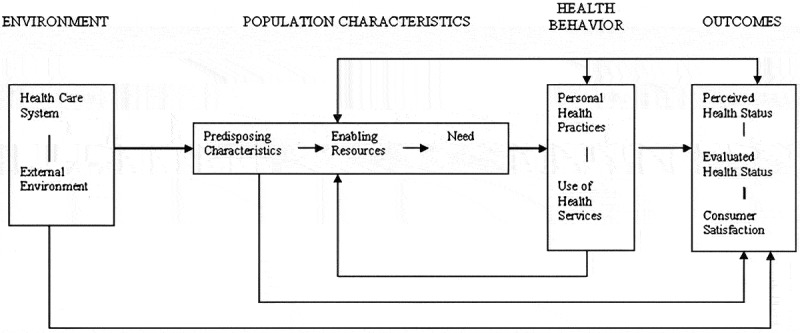
Andersen and Newman behavioural model for health care utilization [18].

Despite these efforts, factors influencing healthcare utilization (HCU) among adults diagnosed with hypertension in Ghana remain poorly understood. The Andersen-Newman Behavioral Model (ANBM) for Health Care Utilization provides a comprehensive framework to study healthcare access, evaluate inequalities, and inform policy [11]. As shown in Figure 1, this model categorizes determinants of HCU into three domains: predisposing factors (e.g. socio-cultural characteristics), enabling factors (e.g. resources to access care), and need factors (e.g. perceived health status). Applying this model to the Ghanaian context can identify barriers and facilitators to healthcare use, guiding strategies to improve access and outcomes for patients with hypertension [11], particularly in resource-constrained settings.

This study evaluated HCU among patients with hypertension to identify critical gaps in healthcare delivery and access for provision of evidence-based recommendations aimed at improving health outcomes.

## Methods

### Study area

This study was embedded within a cluster randomized hybrid effectiveness implementation trial to evaluate the effectiveness of a practical and replicable strategy for implementing Task Strengthening Strategy for Hypertension (TASSH) in Community Health Planning and Services (CHPS) in Ghana [12]. Participants were selected from the TASSH initiative, which aligns with Ghana’s health policy priorities by focusing on improving hypertension management in resource-constrained community settings. The trial was conducted in 70 CHPS zones across three districts – Kintampo North, Kintampo South, and Nkoranza North – in the Bono East Region of Ghana [12]. The study area was chosen for its rural and semi-urban demographics, which reflect the typical settings in which CHPS operates. These districts face a high burden of hypertension [13], limited access to advanced healthcare facilities, and rely heavily on CHPS for primary care delivery. Furthermore, the well-established CHPS infrastructure provided an ideal setting for evaluating the feasibility and scalability of TASSH interventions. Supplementary Table S1 describes the key characteristics of the study area.

### Study design

This cross-sectional study employed a mixed-methods convergent approach, combining quantitative surveys and qualitative interviews, to gain a comprehensive understanding of HCU among stakeholders, including patients with hypertension and community health workers. The qualitative component consisted of individual in-depth interviews and focus group discussions, enabling a detailed exploration of the barriers and facilitators to accessing care. Triangulating quantitative and qualitative data provided a richer understanding of the predisposing, enabling, and need factors influencing HCU.

### Population

The study population consisted of adults aged 40 and above residing in 60 randomly selected CHPS zones out of the 70 CHPS zones for the larger cRCT [12]. On two separate days, participants were screened for hypertension, diagnosed with systolic blood pressure ≥140 mmHg and/or diastolic blood pressure ≥90 mmHg, and referred for care at health centres or district hospitals [12].

### Sample size estimation

The sample size for the quantitative cross-sectional survey was calculated using the single population proportion formula, a commonly applied approach in cross-sectional studies to determine the required sample size for a specified confidence level and margin of error. The formula is given as:

$$n=\;Z^{2}\;*\;p\;*\;\left(1-p\right)/d^{2}$$

Where:

- *n* = required sample size
- Z = Z-score corresponding to the desired confidence level (1.96 for 95% confidence)
- *p* = estimated proportion of the population with the characteristic of interest (healthcare utilization rate, assumed to be 51.4% [14])
- d = margin of error (4% or 0.04)
  $$\;\;\;\;\;\;\;\;\;\;\;\;\;\;\;\;\;\;\;\;\;\;\;\;\;\;\;\;\;\;\;\;\;\;\;\;n={\left(1.96\right)}^{2}\;\times \;0.514\;\times \;\left(1-0.514\right)/{\left(0.04\right)}^{2}$$
  n = 1.9745824× 0.486/0.0016
  n = 599.78

This resulted in an estimated study sample of 600 participants drawn from 60 CHPS zones using a multi-site sampling approach to ensure representativeness across the study area.

For the qualitative component, purposive sampling was employed to ensure the inclusion of diverse perspectives and rich data on healthcare utilization patterns. To achieve this, we conducted 19 in-depth interviews (IDIs) and six focus group discussions (FGDs), which were distributed across the 60 CHPS zones to capture various experiences and contextual factors influencing healthcare utilization.

#### Justification for IDIs and FGDs

IDIs allowed for in-depth exploration of personal accounts and sensitive topics, providing participants with a private setting to discuss challenges and perceptions without the influence of group dynamics. This method was instrumental in uncovering nuanced individual-level insights into predisposing, enabling, and need-related factors influencing healthcare utilization.

FGDs facilitated group interactions, allowing participants to discuss shared experiences and common barriers. They also offered community-level perspectives, emphasizing social and cultural dynamics like using traditional medicine, community norms, and gender-based differences in healthcare-seeking behaviors.

By employing both IDIs and FGDs, the study ensured methodological triangulation, enhancing the reliability and richness of the qualitative data. The IDIs offered detailed individual insights, while the FGDs provided broader community perspectives, contributing to a holistic understanding of healthcare utilization factors.

### Study variables

Referral compliance was the main indicator used to measure healthcare utilization, defined as the percentage of individuals who visited a health facility after the Community Health Officer’s referral during this cross-sectional survey. HCU was coded as a dichotomous outcome: whether the patient visited a health facility or not following the CHW referral. Study participants who visited health facilities after the initial referral by the community health officers were coded as ‘1’, and those who did not were coded as ‘0’.

Variables were categorized according to the ANBM, which included predisposing, enabling, and need factors [15], as shown in Table 1. Predisposing factors considered were age, gender, educational level, and occupation [15]. The enabling factors examined in this analysis included marital status, awareness of hypertension status, access to valid health insurance (which is defined as having an active NHIS membership at the time of data collection), use of traditional medicine, type of referral facility, and the number of lifestyle messages received [15]. For the need factors, we considered hypertension stage and control of blood pressure. Blood pressure was measured directly by trained research assistants. BP records were classified according to standard clinical thresholds: Stage 1 hypertension (systolic 130–139 mmHg or diastolic 80–89 mmHg), Stage 2 hypertension (systolic ≥140 mmHg or diastolic ≥90 mmHg), and severe hypertension (systolic > 180 mmHg and/or diastolic > 120 mmHg). BP control was defined based on whether a participant’s measured BP values fell within recommended treatment targets; systolic blood pressure (SBP) below 140 mmHg and diastolic blood pressure (DBP) below 90 mmHg, in line with widely accepted clinical guidelines [16]. Uncontrolled BP was defined as SBP ≥140 mmHg and/or DBP ≥90 mmHg on repeated measurements, indicating suboptimal hypertension management [17].

**Table 1.** Characteristics of respondents.

Variable	Category	All participants N = 600	Participants who used healthcare N = 439	Participants who did not used healthcare N = 161
**Predisposing Factors**		n(%)	n(%)	n(%)
Age group	40–49 years	107 (17.8)	73 (16.6)	34 (21.1)
	50–59 years	149 (24.8)	110 (25.1)	39 (24.2)
	60–69 years	147 (24.5)	102 (23.2)	45 (28.0)
	70+ years	197 (32.8)	154 (35.1)	43 (26.7)
Gender	Male	207 (34.5)	133 (30.3)	74 (54.0)
	Female	393 (65.5)	306 (69.7)	87 (46.0)
Education Level	None	392 (65.3)	123 (28.0)	56 (34.8)
	Primary/Junior High School	179 (29.8)	7 (1.6)	4 (2.5)
	Senior High School	11 (1.8)	13 (3.0)	5 (3.1)
	Tertiary	18 (3.0)	296 (67.4)	96 (59.6)
Occupation	No occupation	103 (17.2)	76 (17.3)	27 (16.8)
	Has occupation	497 (82.8)	357 (81.3)	130 (80.7)
**Enabling Factors**				
Marital Status	Married	387 (64.5)	277 (63.1)	110 (68.3)
	Not married	213 (35.5)	162 (36.9)	51 (31.7)
Aware of hypertension status	Yes	511 (85.2)	366 (83.4)	145 (90.1)
	No	89 (14.8)	73 (16.6)	16 (9.9)
Valid insurance^a^	Yes	555 (92.5)	417 (95.0)	138 (85.7)
	No	45 (7.5)	22 (5.0)	23 (14.3)
Use of traditional medicine	Yes	102 (17.0)	63 (14.4)	39 (24.2)
	No	498 (83.0)	376 (85.6)	122 (75.8)
Type of referral facility	District hospital	198 (45.1)	198 (45.1)	0 (0.0)
	Health centre	200 (45.6)	200 (45.6)	0 (0.0)
	Other^b^	41 (9.3)	41 (9.3)	0 (0.0)
**Number of lifestyle messages received**				
	One message	195 (32.5)	157 (35.8)	38 (23.6)
	More than one message	405 (67.5)	282 (64.2)	123 (76.4)
Stage of hypertension	Hypertension Stage 1	24 (4.0)	20 (4.6)	4 (2.4)
	Hypertension Stage 2	498 (83.0)	358 (81.5)	140 (87.0)
	Severe hypertension	78 (13.0)	61 (13.9)	17 (10.6)
Blood pressure control	Yes	370 (61.7)	279 (63.6)	91 (56.5)
	No	230 (38.3)	160 (36.4)	70 (43.5)
Access to healthcare (Outcome)	Yes	439 (73.2)		
	No	161 (26.8)		

^a^Having an active NHIS membership at the time of data collection. NHIS is the main scheme within the study setting.

^b^Other include private facility and specialist facilities outside the study area.

### Quantitative data collection and analysis

Two trained research assistants collected the data using a web-based questionnaire, designed with REDCap and administered on Android tablets. The questionnaire was piloted before data collection among 20 participants in a non-study area. No modifications were required; the final tool was used without changes. We first used the ANBM model to pre-select potential determinants. The characteristics of the study participants were described using descriptive statistical analysis. We performed logistic regression analysis to assess the determinants of HCU. We conducted a univariable logistic regression to identify possible determinants at *p* < 0.2. We used a threshold of 0.2 to identify a broader set of potential determinants and ensure that no potentially important variable was overlooked [18]. Significant variables identified in the univariable analysis were included in a multivariable logistic regression model using a backward elimination approach. Multicollinearity was assessed using the Variance Inflation Factor (VIF), with acceptable values (<10) confirming model reliability [19]. Likelihood ratio tests were used to verify model fit. Analyses were conducted using RStudio (version 4.3.1).

### Qualitative data collection and analysis

Nineteen IDIs and six focus group discussions (FGDs) were conducted with patients with hypertension and CHPS community health officers. Participants for both IDIs and FGDs were purposively selected to ensure a diverse range of experiences and perspectives relevant to the study’s objectives. For patients, inclusion criteria were being aged 40 years or older, having a confirmed diagnosis of hypertension, and being recruited as part of the main TASSH Study. Community Health Officers were eligible if they were trained to recruit patients for the main TASSH Study. Recruitment was conducted through CHPS facility records and with the assistance of health officers, who introduced the study to eligible patients during routine visits. The research team then contacted interested participants to provide additional information and obtain written informed consent.

The sample size for the qualitative component was determined based on the principle of data saturation, which is reached when no new themes or insights emerge during data collection. This approach ensures sufficient depth and breadth to explore the research objectives comprehensively. Interviews were conducted in English or Twi, depending on participant preference, to ensure inclusivity and cultural relevance. All sessions were audio-recorded and transcribed verbatim. Data analysis was performed using NVivo for Mac (version 12), employing a thematic analysis approach. SN and DB independently coded the transcripts, identified key patterns, and reconciled discrepancies to ensure rigour and consistency in the findings.

### Ethical considerations

This study was approved by the Kintampo Health Research Centre Institutional Ethics Committee (FWA-00011103: IRB-0004854; Study ID: *KHRCIEC/2021–29*). Participants provided informed consent before the interview, including permission for their audio recordings to be used for the qualitative aspects of the study.

## Results

A total of 600 individuals from the original randomized control TASSH trial participated in this cross-sectional study. This represented ~86% of the original sample of the randomized control trial. As shown in Table 2, study participants were mainly 70+ years and older (~33%) and mostly female (~66%). Most study participants did not have any form of formal education (~66%). Most respondents were married (~65%), aware of their hypertension status (~86%), had valid insurance (~93%), and had received multiple lifestyle messages (~68%). Approximately 17% of the participants had used traditional medicine, with most visiting a health centre (~46%). Most respondents had stage 2 hypertension (83%), and 61.7% had achieved blood pressure control. Following the referral by community health workers, most participants reported using healthcare services (73.2%).

**Table 2.** Characteristics of qualitative respondents.

Characteristics (Patients with hypertension)	FGD participants (N = 51)
**Age (Mean, SD)**	64.0 **±** 12.6
40–49 years	8
50–59 years	14
60–69 years	12
70+ years	17
**Sex**	
Female	34
Male	17
**Highest Educational Level**	
Tertiary	0
Senior High School	6
Junior High School	17
Primary	10
None	18
**Number of years lived with hypertension**	
1–5 years	32
More than 5 years	6
Not reported	13
**Occupation**	
None	12
Trading	9
Farming	30
**Marital status**	
Married	27
Not married	24
Characteristics (Community Health Workers)	IDI Participants(N = 19)
**Age group**	
20–30 years	11
31–40 years	8
**Sex**	
Female	13
Male	6
**Current role**	
Community Health Worker	16
Health Technical officer or Health Field Technician	3
**Years in current role**	
Less than 1 year	2
1 year or more	17
**Number of year(s) involved with screening of hypertension**	
Less than 1 year	8
More than 1 year	11

Table 2 describes the characteristics of the participants who participated in the in-depth interviews and focus group discussions, presented in Table 3. Most of the respondents were females. Most of the patients had lived with the condition between 1–5 years.

**Table 3.** Association between predisposing, enabling, need, other factors and healthcare use among patients with hypertension.

	Univariable logistic regression Odds Ratio (95% CI)		Multivariable logistic regression Adjusted Odds Ratio (95% CI)	
Variable	OR (95% CI)	*P* Value	aOR (95% CI)	*P* Value
***Predisposing Factors***				
**Gender**				
Male	REF		REF	
Female	1.96 (1.54, 2.49)	<0.001	2.12 (1.37, 3.29)	<0.001
**Age group**				
40–49	REF		REF	
50–59	1.31 (0.76, 2.27)	0.328	1.47 (0.78, 2.75)	0.229
60–69	1.06 (0.61, 1.80)	0.843	0.96 (0.53, 1.76)	0.959
70+	1.67 (0.98, 2.83)	0.058	1.97 (1.06, 3.69)	0.101
**Educational Level**				
None	REF		REF	
Primary/Junior High School	0.71 (0.48, 1.06)	0.089	0.85 (0.53, 1.36)	0.490
Senior High School	0.57 (0.17, 2.21)	0.374	0.61 (0.16, 2.63)	0.477
Tertiary	0.84 (0.31, 2.68)	0.752	1.17 (0.36, 4.32)	0.800
**Occupation**				
Has occupation	REF		–	–
No occupation	1.04 (0.0.5, 1.71)	0.876	–	–
***Enabling Factors***				
**Marital Status**				
Not Married	REF		-	–
Married	0.79 (0.53, 1.16)	0.236	–	–
**Awareness of hypertension diagnosis**				
No	REF	–	REF	–
Yes	0.55 (0.30, 0.96)	0.043	0.60 (0.30–1.17)	0.143
**Valid insurance**				
No	REF		REF	
Yes	3.16 (1.70–5.88)	<0.001	3.79 (1.88–7.67)	<0.001
**Number of lifestyle messages received**				
≤ One Message	REF		REF	
> One Message	0.55 (0.36–0.83)	0.005	0.67 (0.40–1.11)	0.125
**Distance to referral facility**				
Less than 30 minutes	REF		REF	
More than 30 minutes	3.56 (2.42–5.29)	<0.001	2.32 (1.47–3.71)	<0.001
**Type of referral facility**				
Health centre	REF		REF	
District hospital	4.41 (2.76–7.30)	<0.001	3.22 (1.1.86–5.72)	<0.001
Other	0.97 (0.15–1.61)	0.243	0.89 (0.50–1.59)	<0.694
**Use of traditional medicine**				
No	REF		REF	
Yes	0.52 (0.34, 0.83)	0.005	0.60 (0.30, 1.17)	0.143
***Need factors***				
**Stages of hypertension**				
Hypertension Stage 1	REF			
Hypertension Stage 2	0.51 (0.15–1.38)	0.228		
Severe Hypertension	0.72 (0.19 - 2.22)	0.588		
**Blood pressure Control**				
Controlled	REF		REF	
Not Controlled	0.75 (0.52–1.08)	0.117	0.98 (0.65–1.49)	0.922

Being female, being 70 years old or older, and having a higher educational level were significant predisposing factors associated with healthcare use in the univariable logistics regression analysis. All enabling factors were significantly associated with HCU, whereas none of the need factors had a significant association with access to healthcare. For each variable, VIF was <2, suggesting absence of multicollinearity in the model (Supplementary Table S1). In the multivariable regression analysis, being older (70+ years), having valid health insurance, distance, and type of health facility were retained as significant predisposing and enabling factors associated with HCU. Female participants had higher odds of utilizing health care than their male counterparts (aOR, 2.32; 95% CI, 1.53–3.54). The odds of HCU were higher in participants aged 70+ years (aOR, 1.97; 95% CI, 1.06–3.69) compared to those aged 40–49 years. Participants with valid health insurance were more likely to use healthcare services (aOR, 4.07; 95% CI, 2.04–8.20) than those without.

Likelihood ratio test (*p* < 0.001) and lower AIC values showed a good fit of the multivariable logistic regression model (see Supplementary Table S2). To further assess explanatory power, we examined pseudo-R^2^ values. The Cox & Snell R^2^ was 0.168, while the Nagelkerke R^2^ was 0.244, suggesting that the model explained approximately 24% of the variance in healthcare utilization among patients with hypertension, relative to the null model.

### Qualitative themes corroborating quantitative findings

Participant responses in the IDIs and Focus FGDs corresponded with the quantitatively identified factors influencing HCU. Male participants’ lower odds of utilizing health care were explained by men being uncomfortable taking anti-hypertensive medication because they worry about some unintended effects of the medication. This sentiment is expressed in the quotation below:

I have heard that when men take the hypertension medication, he becomes weak in bed. So, what that means is that they will not even bother to come to the health at all.

**(IDI, Community health worker, female, 26 years)**

Availability of health insurance, and hence financial cover for costs of the medications for hypertension by the health insurance scheme, was highlighted as a motivation for health care use:

If a person has NHIS that person will not buy the hypertension drug so it will motivate that person to go since the drug is free. **(FGD with patients, Female, 48 years)**

Generally, participants were about four times more likely to travel beyond 30 minutes to receive the required healthcare. They explained that the nearby health centres sometimes do not have the medications available.

So, most patients prefer going to the District Hospital than coming here since mostly we do not have the medications available. **(IDI, Community health worker, female, 30 years)**

### Qualitative themes highlighting barriers and facilitators of health care use

Two main barriers to health care – personal and health systems – emerged from the qualitative data analysis. A recurrent theme in the interviews on personal barriers was a sense amongst interviewees that financial difficulty, use of alternative medicine, and poor health seeking behaviour were seen to be personal barriers for use of health care by patients with hypertension.

Qualitative interviews furthermore highlighted that community members do not have financial resources to enable them to travel to these health centres or district hospitals located far away from their respective communities. Talking about the issue of financial difficulty an interviewee said:

When someone is on hypertension medicine maybe the day, he is supposed to go for the medicine he might not have the money to go for it. So, the person may just have to stay at home and probably die. That is what I have to say. **(FGD with patients, Male, 52 years)**

Others take the drug from district hospital [which is far from the community] so if we ask the person to go to Jema and then take the drug. The person does have any money to go there, they will not bother to go. So, the financial problem is the main cause. **(FGD with patients, Female, 40 years)**

Interviewees expressed the use of other alternative sources for treatment readily available in the community as another personal barrier to HCU for patients with hypertension. These views from interviewees surfaced concerning how the use of alternative source of treatment inhibits health care use for patients with hypertension:

And some of them do say that they heard an announcement in their community about some herbal medicine that can completely cure hypertension and because of that they want to go for the herbal medicine and will not go to the hospital. **(FGD with patients, Male, 49 years)**

Some people say that herbal medicine is better, but the hospital medicine is not good. So, with this belief they are discouraged from coming to the hospital. **(IDI Community Health Worker, Male, 30 years)**

Concerns regarding poor health-seeking behavior were noted during IDIs and FDGs. Interviewees were of the view that, for some reasons, some community members living with hypertension have poor health-seeking behaviour and do not make use of healthcare. Some suggested reasons are highlighted in the quotes below:

Others too just say they do not have time. They say that when they go they will have to join long queues, it will take their time, it means I have to spend the whole day at the hospital. **(FGD with patients, Female, 64 years)**

Yes. So the superstitious beliefs do come to play here and it does not help for them to go to the health centres for care. **(IDI Community Health Worker, Female, 32 years)**

Interviewees also highlighted some health system-level barriers that hinder HCU for patients with hypertension. Repeated themes in the interviews regarding personal barriers were the perception among interviewees that erratic drug supply was a hindrance to the utilization of healthcare services. Views among interviewees are highlighted below:

The issue is that when we go they will write the medication for us to go and buy since they do not have it at the hospital. And there is a financial difficulty, so I choose not to go anymore because anytime I go, they write the medicine for me to go and buy. **(FGD with patients, Female, 54 years)**

The doctors too are to be blamed when we go to the doctor and the medicine is not available, they will write for us to go and buy at a particular place and this serve as a hindrance for us to go to the hospital. **(FGD with patients, Male, 50 years)**

A further issue was that there was only partial health insurance coverage for individuals who had used health services. Interviewee suggestions are highlighted by the quotations below:

Sometimes health insurance cover the medications and some other times we pay. They give us the medication but there are few expenses. **(FGD with patients, Female, 45 years)**

To go there without money … that is why I asked about the health insurance, probably I may go with health insurance and might still be asked to pay for some other things so without money it will become a problem. **(FGD with patients, Female, 65 years)**

When the issue of facilitators seeking health care was explored, three facilitators emerged from the analysis of the qualitative data: changes in hypertension management policies to allow for medication to be given at peripheral facilities, strengthening home visits, and improving communication between health workers and patients:

If possible and you know the number of patients, then the medicine can be given to the CHPS compound here so that we can get to take it. After taking it for a while we can go to the District Hospital for review. **(FGD with patients, Female, 58 years)**

Participants clearly articulated that improving communication between health workers and patients will enhance access to care for patients with hypertension. Patients were of the view that they mostly do not know they have hypertension until they are screened and informed on what to, indicated that if the nurse does not tell them what to do, they will not know what to do to address their high blood pressure:

We all thought we were fine until we were screened, we were told we have hypertension. So, if you could be telling us regularly what we should do, this will be helpful. **(FGD with patients, Female, 48 years)**

Table 4 summarizes quantitative and qualitative findings across the ANBM domains. Qualitative insights explained underlying reasons for quantitative findings such as men’s reluctance, financial challenges, and perceptions of service availability.

**Table 4.** Joint display of quantitative and qualitative findings on enabling factors influencing healthcare utilization.

ANBM Domain	Quantitative Findings	Qualitative Findings	Interpretation
**Predisposing factors**			
Gender	Women were more than twice as likely to use healthcare as men	*There was indication of males generally not taking medication because of side effects of medication.*	Gender differences reflect higher health-seeking behaviour among women and reluctance among men due to fears of side effects.
Age	Older adults (70+ years) had higher odds of healthcare use.	FGDs suggested that older adults, especially women, were more motivated to seek care because of perceived vulnerability to complications.	Age increases perceived need and willingness to seek care but also intersects with financial and mobility barriers.
Education	Education was not significantly associated with healthcare use in adjusted models.	No clear qualitative evidence linking education to utilization emerged.	Education does not appear to directly drive utilization in these setting; other enabling factors (insurance, facility type) may play a stronger role.
**Enabling Factor**			
Health Insurance (NHIS)	Patients with valid insurance were four times more likely to use healthcare.	Participants indicated if patients are with health insurance, then they seek healthcare.	NHIS reduces cost barriers, but incomplete coverage and hidden costs remain obstacles.
**Distance to Facility**	Longer travel time (>30 minutes) was associated with higher odds of utilization	Participants reported challenges with traveling longer distances to use health services.	Patients often bypass nearby facilities for better resourced hospitals, but distance interacts with affordability and can still discourage use.
**Type of Facility**	Patients referred to district hospitals were over three times more likely to use care than those referred to health centres	*Patients preferred using district hospitals because they get the needed care.*	District hospitals are perceived as more reliable (staff, medicines), reinforcing bypass behaviour.
**Need Factor**			
**Hypertension stage**	Not significantly associated with healthcare use.	No strong qualitative evidence linking disease severity to utilization emerged.	Perceived need does not appear to drive utilization as strongly as enabling factors (insurance, distance, facility).
**Blood pressure control**	Not significantly associated with healthcare use.	Some respondents reported feeling ‘fine’ despite elevated BP, reducing motivation to seek care.	Patients may underestimate their need for care; awareness campaigns could bridge this gap.

## Discussion

To our knowledge, this study is among the first to apply the Andersen’s Behavioural Model to explore HCU in Ghana. This study analyzed and triangulated qualitative and quantitative data on predisposing, enabling and health-system-related factors affecting HCU among patients with hypertension. Gender emerged as the only predisposing factor, while enabling factors included having a valid health insurance, the distance to care, and the type of referral. No need factors significantly determined HCU. Personal barriers included financial difficulties, reliance on traditional medicine, and poor health-seeking behavior, while systemic barriers consisted of anticipated delays, erratic medication supplies, and limited insurance coverage. Facilitators of HCU included health policy changes, home visits, and improved communication between patients and providers.

In our study, 73% of patients with hypertension used healthcare services. This rate is relatively high compared with global estimates of healthcare utilization among individuals with chronic cardiovascular conditions. For example, real-world analyses of treatment patterns for pulmonary hypertension in the United States have shown that although nearly all patients required ongoing clinical follow-up, only about 55–65% consistently used healthcare services [20]. A study conducted in Dar es Salam found low utilization of health services following hypertension screening [21]. Within Ghana, previous studies have documented lower levels of HCU, suggesting that patient engagement and health system responsiveness related to TASSH have positive effects [4,22]. Moreover, 93% of the study participants had valid health insurance, significantly enhancing their financial access to healthcare. Participants with valid health insurance were four times more likely to utilize healthcare services than those without coverage. The NHIS is the main and most widely used health insurance scheme among community members. This underscores the critical role of Ghana’s NHIS in reducing financial barriers to care [8,23]. Despite this progress, challenges persist. For instance, qualitative findings suggest that some hypertensive medications are only partially covered under the NHIS and this imposes additional financial burden on patients. This limitation reflects broader systemic issues, as inadequate healthcare financing continues to hinder the ability of health systems in Ghana and other countries to fully address the population’s health needs across the life course [24]. Addressing these gaps will require sustained investment and policy efforts to ensure comprehensive coverage and equitable access to healthcare services.

Women were more than twice as likely to utilize health care as men, in line with previous observations in Ghana and elsewhere in SSA [25,26]. This disparity aligns with evidence suggesting that women generally exhibit higher levels of health-seeking behavior and compliance with medical advice compared to men [27–31]. Gender differences significantly influence access to and the quality of healthcare services [31]. Women, for instance, tend to prioritize their health more actively, often engaging in preventive care and scheduling regular medical checkups. This behavior may be shaped by societal norms that emphasize the importance of women’s health, particularly in their roles as caregivers within families, as well as biological vulnerabilities that expose them to unique health challenges, such as reproductive health issues [32]. In contrast, men are more likely to delay seeking medical attention until the condition becomes severe, contributing to higher incidences of preventable illnesses and complications among men [33]. This reluctance to seek care may stem from various factors, including work-related time constraints, cultural perceptions or masculinity that discourage seeking help, and limited awareness of the benefits of preventive care [34]. Addressing these barriers requires targeted interventions, such as workplace health programmes and campaigns to normalize proactive health-seeking behaviors among men.

Personal barriers to HCU were financial difficulties, reliance on alternative medicine, and poor health-seeking behaviour, possibly related to financial difficulties. On the healthcare system’s side, barriers included anticipated delays, erratic medication supplies, and partial coverage by health insurance. These findings align with observations by Koduah and colleagues, who identified unhealthy lifestyles, poor health-seeking behavior, and inadequate health system capacity as key impediments to achieving universal health coverage for patients with hypertension [35]. This highlights the urgent need for targeted interventions to address personal and systemic healthcare barriers. Improving the health system by ensuring timely care, reliable medication supply, and comprehensive insurance coverage can directly mitigate health system obstacles. This, in turn, has the potential to alleviate personal barriers, such as financial strain, and promote healthier behaviors. Addressing these interconnected challenges is critical to enhancing healthcare utilization and achieving better health outcomes for patients with hypertension in Ghana.

Participants in our study were about 4 times more likely to use a district hospital than a health centre. This can be attributed in part to the current national NCD policy, where CHPS compound staff are not permitted to dispense hypertension medication [36] and in part to the expected availability of a medical doctor and antihypertensive medication as compared to peripheral facilities [22]. Additionally, due to accreditation challenges, some health centres do not receive reimbursement for providing antihypertensives. Consequently, community members must travel longer distances to access healthcare, which can be particularly challenging for those unable to afford transportation. Several ongoing trials at the community level in Ghana aim to address these issues by ensuring the restocking of hypertension medications [37,38]. While these initiatives promise to improve hypertension management at the community level, their scalability remains a critical challenge. Expanding these programs would reduce travel burdens, enhance access to care, and ultimately improve hypertension management and outcomes for patients in underserved areas.

In our study, we identified changes in the current NCD policy, home visits, and improvements in communication to facilitate healthcare use among patients with hypertension. Community health workers have demonstrated their potential to play a crucial role in hypertension management in Ghana. Community-based interventions such as the Community-based Hypertension Improvement Project (ComHIP) have effectively improved hypertension control at the community level [37]. Understanding community perceptions and practices, as well as the need for community-specific interventions to improve awareness, treatment, and control of hypertension [39,40] may enhance home visits and communication between community health workers and patients with hypertension. These findings highlight the potential for community health workers to play a crucial role in managing hypertension in Ghana.

We used the three domains of the ANBM to enhance our understanding of the factors influencing healthcare utilization among patients with hypertension. This allowed us to identify potential barriers to care and develop targeted interventions to improve healthcare outcomes for patients with hypertension by contextualizing predisposing, enabling, and need factors. Additional components of the model, however, were not included in this study. For instance, data on health beliefs and perceived need factors were not collected. To broaden coverage and thoroughly examine the factors related to healthcare utilization, it is recommended that the ANBM be utilized in conjunction with other frameworks [41].

The study’s strengths include its integration within the TASSH trial and the triangulation of multiple data sources, providing a comprehensive understanding of healthcare utilization among patients with hypertension. The integration of quantitative and qualitative data, guided by the Andersen Behavioural Model, provided a common interpretive framework. Differences between data strands were seen as opportunities to enhance understanding rather than as discrepancies to be fixed. The study’s findings will be helpful in initiating discussions on potential barriers to universal health coverage for patients with hypertension in Ghana. However, we recognize that using self-reported data on HCU can be prone to reporting bias and that our findings cannot easily be generalized to patients with severe health impairments or other countries with their own healthcare system challenges. Additionally, due to the cross-sectional nature of this study, we cannot rule out the possibility of reverse causation. Furthermore, our study’s linkage to a larger trial promoting care-seeking may have contributed to higher utilization. We did not collect data on their occupation and marital status from the community health workers. Thus, data is not shown.

## Conclusions and implications for strengthening health services

Guided by the ANBM for healthcare utilization, we assessed factors affecting HCU among patients with hypertension in a rural setting. Gender was the only predisposing factor. Enabling factors included valid health insurance, distance to care, and referral type. Personal barriers were financial issues, reliance on traditional medicine, and poor health-seeking behavior. Systemic barriers included anticipated delays, erratic medication supplies, and limited insurance coverage. HCU facilitators implemented health policy changes, conducted home visits, and enhanced communication between patients and providers. Interventions should address gender and age disparities, expand health insurance, and improve district hospital access. Policymakers must also tackle barriers such as medication supply and financial constraints. Future research should evaluate quality of care to improve hypertension management.

## Supplementary Material

Supplementary Tables File.docx

## Data Availability

The data will be made available at a reasonable request to the corresponding author.
