# Supplementary material for: Determinants, barriers, and facilitators of healthcare access for patients with hypertension in rural Ghana: applying the Andersen-Newman model of healthcare utilization
Source: Glob Health Action. 2025 Dec 22;18(1):2599567. doi: 10.1080/16549716.2025.2599567 (PMC12724238; doi:10.1080/16549716.2025.2599567)
Supplement: Supplementary Tables File.docx [file ZGHA_A_2599567_SM3755.docx]

Supplementary Table 1

Additional file 3: **Table S3** Test of multicollinearity using generalised variance inflation factor for logistic regression model (model 3)

| **Variable** | **GVIF** | **DF** | **GVIF^(1/2*DF)** |
| --- | --- | --- | --- |
| Sex | 1.16 | 1 | 1.08 |
| Age group | 1.25 | **3** | 1.04 |
| Education | 1.40 | 3 | 1.06 |
| Hypertension awareness | 1.23 | 1 | 1.11 |
| Use of traditional medicine | 1.09 | 1 | 1.04 |
| Type of Health Facility | 1.27 | 2 | 1.06 |
| Valid Health Insurance | 1.13 | 1 | 1.06 |
| Number of lifestyle messages received | **1.27** | 1 | 1.13 |
| Distance to referral facility | 1.24 | 1 | 1.11 |
| Blood pressure Control | 1.03 | 1 | 1.03 |

GVIF=Generalized Variance Inflation Factor, DF=Degree of Freedom

Supplementary Table 2

Additional file 2: **Table S2** Goodness of fit test

| Model | DF | AIC | Deviance | P-value |
| --- | --- | --- | --- | --- |
| 1 | 20 | 620.6 | 290.3 |  |
| 2 | 16 | 619.9 | 294.0 | 0.121 |

DF=Degree of Freedom, BIC=Bayesian Information Criterion.

Supplementary Table 3

Additional file 3: Description of key characteristics from the study area

| **Characteristic** | **Kintampo North Municipality** | **Kintampo South District** | **Nkoranza North District** |
| --- | --- | --- | --- |
| Population size | 120,000 | 90,000 | 70,000 |
| Main economic activities | Farming and petty trading | Farming (yam, maize, cassava) | Farming (yam, maize), livestock rearing |
| Literacy rate (%) | 68 | 61 | 65 |
| Health facilities | 1 District Hospital, 8 Health Centres, 38 CHPS Compounds | 1 District Hospital, 3 Health Centre, 29 CHPS Compounds | 3 Health Centres, 24 CHPS Compounds |
| Top 3 causes of outpatient morbidity | Malaria, Acute Respiratory Infection (ARI), Hypertension | Malaria, Acute Respiratory Infection (ARI), Diarrhoea | Malaria, Acute Respiratory Infection (ARI), Hypertension |
